# Relationship between Language Dominance and Stimulus-Stimulus or Stimulus-Response Inhibition in Uyghur-Chinese Bilinguals with an Investigation of Speed-Accuracy Trade-Offs

**DOI:** 10.3390/bs9040041

**Published:** 2019-04-18

**Authors:** Ruilin Wu, Esli Struys, Katja Lochtman

**Affiliations:** 1 Centre for Linguistics, Department of Linguistics and Literary Studies, Vrije Universiteit Brussel, 1050 Brussels, Belgium; Esli.Struys@vub.be (E.S.); Katja.Lochtman@vub.be (K.L.); 2 Center for Neurosciences, Vrije Universiteit Brussel, 1050 Brussels, Belgium

**Keywords:** bilingual language dominance, Stimulus-Stimulus inhibition, Stimulus-Response inhibition, speed-accuracy trade-off

## Abstract

The effect of bilingualism on inhibition control is increasingly under ongoing exploration. The present study primarily investigated the effect of within bilingual factors (i.e., dominance types of Uyghur-Chinese bilinguals) on a Stimulus-Stimulus task (Flanker) and a Stimulus-Response task (Simon). We also compared the bilinguals’ performance on each type of cognitive control task in respect to a possible trade-off between speed and accuracy. The findings showed no explicit differences on performance in response time or accuracy among balanced, L1-dominant and L2-dominant bilinguals but balanced bilinguals demonstrated a significant speed-accuracy trade-off in the overall context switching between non-conflict and conflict trials in both cognitive control tasks where monitoring process is highly demanded. Additionally, all bilinguals across all language dominance types showed a trade-off strategy in inhibition during a Stimulus-Stimulus conflict (flanker task). This evidence indicates that the differences of within bilinguals in cognitive control could lie in the monitoring process, while for all bilinguals, inhibition during a Stimulus-Stimulus conflict could be a major component in the mechanism of bilingual language processing.

## 1. Introduction

In research on bilingualism, numerous studies have shown an ongoing interest in the exploration of the relationship between bilingual experience and executive functioning. Faced with the challenge of two competing languages, bilinguals continuously need to process the co-activated representations of target and non-target languages. There are different models accounting for the bilingual language control. Some researchers (e.g., [1]) claim that the interference of the irrelevant language is inhibited via a top-down inhibitory control mechanism. This constant management of multiple languages is proposed to develop and enhance the domain-general executive control (see for an overview [2,3]). However, Dijkstra et al. [4,5] argue that the selection of relevant language is processed via a bottom-up mechanism encapsulated in a word identification system. Paap et al. [6] have questioned the bilingual superior performance in executive functioning and argue that there is either no relationship between bilingualism and executive functioning or that other factors are contributable to the bilingual advantage when specific conditions are matched. By taking a review of previously conducted tests, it is found that 17% of studies reported the findings of a bilingual cognitive control advantage in non-linguistic tasks that involve the monitoring of conflict and the resolution of conflicting information [6]. That is, bilingual advantages in the Simon [7,8] and flanker task [3,9,10,11,12] are occasionally reported. However, there are 80% reported null results [6] and a recent meta-analytical review [13] revealed the lack of sufficient support for bilingual advantage. Given the inconsistency in findings, the investigation of cognitive correlates of bilingualism is more carefully explored when specific variables are controlled for. Recently, apart from viewing bilinguals as a homogeneous group to compare with monolinguals, a growing number of studies [14,15,16] have suggested that certain within-bilingual factors such as sociolinguistic ones or bilingual dominance types be attributable to the so-called bilingual advantage.

### 1.1. Previous Studies on the Importance of Sociolinguistic Factors in Bilingual Advantage in Regional Minority Languages

One confounding variable within bilingualism that is proposed to be relevant is the sociolinguistic setting of bilinguals [17]. In the research of Blom et al. [14], Frisian-Dutch and Limburgish-Dutch bilinguals who acquire a regional language in addition to a national one and a group of immigrant bilinguals exposed to Polish and Dutch were separately compared with a Dutch monolingual group. Though Frisian, Limburgish and Polish are all minority languages, they vary greatly in the sociolinguistic domain. A regional language such as Frisian has taken up a wide use in the public media, administrative and educational domains, whereas the exposure to an immigrant language such as Polish is primarily sourced from the home context. The results of a flanker task demonstrated that a cognitive effect was found in the Frisian-Dutch language group but it was less robust in Limburgish bilinguals. In contrast, the bilingual advantage was not present in the subgroup of immigrant bilinguals who had a low proficiency of the minority language Polish. These findings indicate that the sociolinguistic setting plays a role in the bilingual advantage on the condition that the level of language proficiency is as well taken into consideration. Therefore, it is necessary to control the sociolinguistic background and keep it matched when the effect of bilingualism on executive functioning is explored. Additionally, the varying results in the flanker task between Frisian-Dutch and Limburgish-Dutch bilinguals suggest that a more considerable exploration and investigation should be conducted to the effect of regional minority language bilingualism on the cognition ability of inhibition.

However, in fact only a limited number of studies have tapped into this aspect. Among previous studies that attach importance to bilinguals in a regional minority language, their cognitive control over interference was either tested with the Stimulus-Stimulus type inhibition, such as the flanker task, the Stroop task or with the Stimulus-Response pattern, including the Simon task. This taxonomy stems from the Dimensional Overlap Model [18] and it specifies the different mechanisms underlying the compatibility effect. That is, the cause of the conflict effect is distinct, one deriving from the overlap of Stimulus-Stimulus and another from that of Stimulus-Response dimensions. For instance, in the flanker task (Stimulus-Stimulus inhibition), there is an overlap between the two dimensions of stimulus: the orientation of the central arrow and the orientation of the surrounding arrows, while in the Simon task (Stimulus-Response inhibition), the overlap exists between the task-irrelevant stimulus dimension (the location of the stimulus: left or right) and the response dimension (the location of the response key: left or right).

In the studies of regional minority language bilinguals, it is noteworthy that their cognitive control in inhibition were usually exclusively evaluated by one type of interference tasks, either Stimulus-Stimulus or Stimulus-Response incompatibility (e.g., [15,19]) and the findings of cognitive benefit in inhibition are inconsistent and variable across flanker type interference and Simon type interference. Costa et al. [3] administered various low and high monitoring versions of the flanker task and compared Catalan-Spanish bilinguals and Spanish monolinguals. Their results reported that a bilingual advantage was significant across two high demanding conditions compared to the low monitoring conditions and especially the magnitude of effect was larger when the congruent and incongruent conditions were equally proportional. By contrast to Costa et al. [3] findings, Antón et al. [19] found that Basque-Spanish bilinguals performed similarly compared to Spanish monolinguals in all of the conflict, alerting and orienting conditions in Attentional Network Task (ANT) where the test of conflict detection and control is equivalent to a flanker task. In another study [20] with a group of Basque-Spanish bilinguals, no bilingual advantage was reported for both the numerical and classic Stroop tasks in terms of accuracy and reaction time. However, the evidence from the Simon task in the study conducted by Antoniou et al. [21] demonstrated a positive effect on cognitive control with a control over the factor of language proficiency in the weaker language when comparing the bilinguals (bidialectals) speaking Cypriot Greek and Greek and the monolinguals in Greek. Unlike the preceding regional minority language pairs, these two varieties of the same Greek language are in a diglossic context where the language distance is close but the language use is clearly distinct with one being vernacular and another used in a formal environment. Gathercole et al. [15] administered a Simon task on Welsh-English bilinguals from 3 years old to over 60 years of age and they grouped the bilinguals into three types in accordance with their home languages, that is, Welsh dominant, balanced, English-dominant. The results revealed few findings towards a cognitive benefit but with the older adults, all bilinguals showed a superior performance and in case of the children’s group, bilinguals speaking Welsh as their home language outperformed the ones with English as their home language. This brief review indicates that little attention was paid to the comparison between the two different patterns in inhibitory control mechanism within the homogeneous groups of regional language bilinguals.

### 1.2. Assessing Bilingual Language Dominance

In the reviewed literature, the studies with positive findings show that bilingual proficiency is another confounding variable which mediates the effect on cognitive advantage [14,21]. Being bilingual does not imply being equally proficient in each acquired language. Bilinguals may develop a balanced language proficiency in both languages or they could have a better language knowledge in one of them [22]. Bilingual proficiency can be interpreted as respectively evaluated language proficiency of each language or as a relative proficiency of two languages for an individual. This is unique to bilingualism, because monolinguals have language proficiency but no relative balance or non-balance in the proficiency of more than one language. Following this line of reasoning, it is more meaningful to explore the variable of degree of bilingual proficiency in terms of balance or non-balance, since individual bilingual language proficiency can be situated on a continuum of language ability [23] and bilinguals can be subdivided into different types of bilingual dominance.

A review of studies involving the measures of degrees of bilingualism (e.g., [24,25,26,27]), has revealed a lack of uniform assessment that is indicative of dominance of bilingualism and different researchers evaluate and compute relative bilingual language proficiency by using different measures [28]. Similarly, the assessment instruments for bilingual dominance are also divergent in the studies on the effect of degree of bilingualism on inhibition control in tasks involving the interference suppression. In some studies, the ratio of bilingual first language proficiency to the second one was determined by performance on language reception tasks, such as receptive grammatical knowledge [29] and linguistic ability of receptive vocabulary, that is, the Peabody Picture Vocabulary Test [30,31,32], while others defined the bilingual balance based on the verbally expressive vocabulary tasks [33]. There are studies that use both expressive and receptive language ability in vocabulary [34] or on a combination of morphologically expressive and lexically receptive tasks [35] and on a set of both language comprehension and verbal production tests [36]. Additionally, a variety of other general factors are also used to index the relative dominance of bilinguals: the use of home language [37], a self-assessment of language recognition and expression skills as well as external ratings according to school grades [38] or self-evaluation of literacy skills and frequency of language use, collected from well-designed questionnaires [12].

Basically, the assessment tools in the previous studies can be approximately summarized into lexical and grammatical knowledge tests on the one hand and self-reported questionnaire-oriented measures on the other. In the reviewed literature, a language questionnaire is primarily adopted by the studies in which the bilinguals or multilinguals are native speakers of the minority language [37,38], except for the Frisian-Dutch bilinguals in the study of Bosma et al. [35]. For studying the bilinguals whose language is not spoken by the researchers, it is usually reliable to use a valid self-evaluation questionnaire. In the present study, the Language Experience and Proficiency Questionnaire [39] is used to delineate and capture the comprehensive and overall aspects of language in terms of language proficiency, language preference, acquisition age, frequency of use and so forth.

To our knowledge, the present study is the first to determine the relative dominance of bilinguals according to the respective ratings of proficiency of linguistic skills and language preference. The balance of bilingualism is highly dependent on the individual. Previously, language preference for language use in computational settings was found to be a predictor to the relative dominance of bilinguals [40]. It indicates that language preference is a reliable indicator to assess bilingual individual’s affective awareness towards language performance. 

Besides, among the reviewed literature involving the conflict resolution tasks, three studies working with regional language bilingualism have explored the role of relative dominance of bilingualism on Stimulus-Stimulus tasks [35,37,38], while one study by Gathercole et al. [15] was tested with the Simon task, indicating that with home language as the indicator of degrees of bilingual dominance, the preschool group of bilinguals dominant in regional language, Welsh, outperformed the balanced bilinguals and the ones dominant in national language, English. On the contrary, the findings with Stimulus-Stimulus tasks are inconsistent. The findings of Bosma et al. [35] demonstrated that language balance of Frisian-Dutch bilinguals showed no effect on the inhibitory control tested by the flanker task but a positive effect on selective attention tested by the Sky Search task. Similarly, Videsott et al. [38] found that interference suppression was not predicted by the relative language competence of multilinguals in an ANT task but attentional control was better in multilinguals with a higher linguistic competence. However, Gathercole et al. [37] reported a cognitive benefit in Welsh-English balanced bilinguals with the measure of the Stroop task when tested in English.

### 1.3. Present Study

A recent study conducted by Paap et al. [41] has investigated the distinction between cognitive control tasks with Stimulus-Stimulus or Stimulus-Response conflict in bilinguals speaking various languages in addition to English. Their findings suggest that the spatial Stroop task and the Simon task may rely on a shared inhibitory control mechanism but that this general inhibitory control ability is not employed in the bilingual language selection process. This study is the first to test these two task types in considering the bilingual language experience in dominance. It is also important that this study found the scores on the flanker task to be different from the other interference tasks, so this is highly relevant for our study. Especially, this task-related variable was rarely taken into consideration when studying bilinguals speaking a regional language. Moreover, relative language dominance of bilinguals has been gradually considered as a confounding variable when executive functioning was measured. However, there is a lack of studies to address to what extent bilingual language dominance predicts the minority language bilingual’s performance on the two constructs, that is, the Stimulus-Stimulus type and the Stimulus-Response type of inhibition control.

The present study is to control for the sociolinguistic factor of bilingualism by focusing on Uyghur-Chinese minority language bilinguals. Ethnic Uyghur is one of minorities living in their own autonomous region in the north-western part of China, Xinjiang Uygur Autonomous Region. Among all the ethnic minority languages in Xinjiang, Uyghur language is a regional language at the administrative, educational and community contexts. In the autonomous ethnic minority region, both the national language Chinese and the indigenous language Uyghur are recognized as official and legitimate languages that can be applied concurrently not only for official and social public occasions but also as languages of instruction at local schools. Since 2000, the government of Xinjiang Autonomous Region has begun to actively promote bilingual education and therefore the education trajectory for ethnic Uyghurs varies a lot. For instance, they may attend Uyghur language instruction schools at elementary level and then transfer to Uyghur-Chinese bilingual classes in middle school and then they may be admitted to Chinese language instruction schools in high school. It is indicative that the experience of Uyghur-Chinese bilinguals may vary due to the possibility of divergent combinations of education trajectories in the school context.

Across previous studies on bilingual advantage, a study targeting regional language bilinguals with the specific language pair of Uyghur and Chinese is lacking. It is noted that the languages of Uyghur and Chinese typologically and morphologically differ from each other. Uyghur belongs to the Turkic language family, whereas Chinese is one of Sino-Tibetan languages. Uyghur is an agglutinative language with the feature of morphological changes and its syntactic order is subject-object-verb. In contrast, a Chinese morpheme has no inflection and its grammatical order is subject-verb-object. However, as Uyghur is in the Turkic language family, the Turkish language shares similarities with the Uyghur language. A recent study conducted by Oschwald et al. [42] explored the role of cross-language distance in the executive function by the investigation of German-Turkish bilinguals. The findings in their study demonstrated that language similarity has no effect on modulating the executive functioning processes of inhibition, monitoring, mixing, shifting and working memory. 

With the focus on the Uyghur minority language bilingual population, the first research question in the current study is to examine whether the inhibition control measured in two patterns of conflict resolution tasks is moderated by the language dominance type of Uyghur-Chinese bilinguals. We hypothesize that the balanced bilinguals have a better performance in each cognitive control task than the dominant bilinguals, for the activation of each language is relatively equal which demands more selective control, compared to the non-balanced bilingual individuals with one much less active language [43]. Nevertheless, an alternative assumption is worth noting. Paap’s [44] controlled-dose hypothesis implies that the required executive functioning may dissipate when bilinguals have achieved a balanced proficiency in their two languages, which would result in a superior performance for the L1- or L2-dominant bilinguals on the two interference tasks.

Another research aim is to explore which underlying mechanism of the interference suppression tasks best accounts for the bilingual advantage measured by the flanker task and the Simon task. To be noted, the Stroop task is not selected to represent the Stimulus-Stimulus pattern, because the classic version of this task is highly dependent on language and cannot be purely counted as a non-linguistic task [45]. In the previous studies, an important finding is that these two types of inhibition mechanism are correspondent to different components in language processing. Studies revealed that for bilinguals, conflict resolution performance in a Stimulus-Stimulus task (a non-linguistic Stroop task) correlated to their language comprehension process of visual word recognition [46] or to auditory word recognition process [47]. Additionally, bilingual performance in speech production has been found to be linked to the Stimulus-Response inhibition (Simon task) [48] or the Stimulus-Stimulus inhibition (flanker task) has been reported to be facilitated by a language switching context [49].

In light of the previous findings, it is possible to deduce that bilinguals are to some extent sensitive to a Stimulus-Stimulus inhibitory mechanism. In respect to comparing these two patterns of cognitive control tasks, Blumenfeld & Marian [50] conducted two experiments that only differ in terms of bilingual language profiles. Their evidence weakly supported that bilinguals with two international and widely spoken languages, English and Spanish, showed a bilingual advantage in the Stroop type inhibition compared to the Simon type inhibition. The better performance in Stimulus-Stimulus incompatibility only occurred when measured in terms of accuracy and efficiency scores in the first experiment but it was not observed in response times of interference suppression for both experiments. Moreover, the findings in Paap et al.’s study [41] revealed the absence of a bilingual advantage in both types of cognitive control tasks. To be noted, their results revealed that the mechanism underlying a flanker type inhibition was distinct from spatial and vertical Stroop tasks and the Simon task. Therefore, for dominance subsets of regional language bilinguals in the present study, we are interested to explore whether there will be a difference in the inhibitory control between the flanker task and the Simon task. Our assumption is that each dominance subset of bilinguals may show differences between the flanker task and the Simon task and the differences are expected to be found in the strategy use for trading-off between response time and accuracy rates, in line with Struys et al. [51].

More importantly, concerning the comparison between the two cognitive control tasks, the approach that results are separately interpreted from one dimension of response time or accuracy cannot fully reveal the actual performance, for there exists an influence from a strategic tendency for either focusing on speed or on accuracy which was named as speed-accuracy trade-off [52]. This trade-off strategy was found in cognitive control tasks, for instance in Simon or flanker tasks (e.g. [53,54]. Thus, in our present study, we aim to compare bilingual cognitive performance by examining the correlation between reaction time and accuracy [51]. To be noted, the evidence from the study by Frühholz et al. [55] shows that the incompatibility arising in the flanker task causes a stronger behavioural conflict effect than the incongruency generated in the Simon task. Given that more efforts may be exploited to resolve the conflict inducing a stronger interference effect on a behavioural level, we hypothesize that all bilingual groups demonstrate a strategic tendency for either a faster reaction at the cost of lower accuracy or a higher accuracy rate compensated by a slower response in the flanker task as opposed to the Simon task. We hypothesize that all bilingual groups demonstrate a strategy tendency for either a faster reaction at the cost of a lower or a higher accuracy rate compensated by a slower response in the flanker task as opposed to the Simon task.

## 2. Materials and Methods

### 2.1. Participants

This study was part of a larger research project. Uyghur-Chinese bilingual young adults were recruited from the undergraduate student population of universities in Xi’an, Shaanxi province in China. 159 participants filled out the LEAP-Q when we collected the data for evaluating their bilingual language profile but 66 out of them gave no informed consent for participation in the cognitive part of the project. In total, 93 participants (34 males and 59 females) with a mean age of 19.59 years (*SD* = 1.36) gave an informed consent prior to the experiments and took part in this study. All participants were admitted into university through the National Higher Education Entrance Examination (a standardized academic examination every year) and they had normal or corrected to normal vision. Additionally, concerning the sociolinguistic context of participants at the moment of the experiment, they all studied in a university where Chinese is the medium of instruction. They self-reported that their language speaking, listening, reading or learning abilities were not impaired. Before starting bachelor programmes in the city of Xi’an, all participants were indigenous habitants in Xinjiang Uyghur Autonomous Region of China where Uyghur is a regional language with an officially recognized status and they were all native speakers of Uyghur.

#### 2.1.1. Language Profiles

To get access to their language profiles, an adapted version of the language experience and proficiency questionnaire (LEAP-Q) was used [39]. In the daily language context, the mean frequency of their exposure to the Uyghur language (48%) and the Chinese language (41%) is to some extent similar and they also have the chance to be exposed to other languages (11%). For the subsequent assessment of the degree of bilingual proficiency, participants self-evaluated their language literacy skills respectively for Uyghur and Chinese on the basis of a ten-point Likert scale ranging from 0 (= no knowledge) to 10 (= perfect command). The data showed that the average score for the Uyghur language proficiency of the participants (*N* = 93) is 8.84 (*SD* = 1.37) and that for Chinese is 7.83 (*SD* = 1.40). Additionally, language preference for using each language in certain contexts such as conversation or reading and so forth, was also self-rated by the participants based on a percentage frequency ranging from 0% (= no preference) to 100% (= highest preference). The results revealed that the mean probability of participants preferring to use Uyghur is 48% (*SD* = 16.57) and for Chinese is 44% (*SD* = 14.83) and for other languages is 8% (*SD* = 7.52).

#### 2.1.2. Measures of Bilingual Language Dominance

The bilingual language preference for cognitive activities such as the bilingual performance in number processing or thinking to oneself is correlated with the variable of the bilingual dominant language (measured through the self-rated language proficiency of each language) or the length of exposure in the additionally acquired language [40]. Given that there is an intrinsic connection between language preference and language dominance in bilinguals, the measures for assessing the language dominance of participants in the present study were based on a combination of self-evaluated language proficiency and language preference. 

Specifically, in the first step, the original scores of language proficiency and preference were transformed into standard scores, because these two index factors were rated respectively on different scales, a ten-point scale and a percentage scale. With standard scores, it allows us to compare these two scores with the same unit. Then, the next step was to calculate the ratio of Uyghur (L1) and Chinese (L2) to indicate the relative proficiency. However, to be suitable for calculating a ratio, the standard scores needed a further formula. The reason is that if the raw score is below the mean, its standard score is correspondingly a negative number. Moreover, if the raw score equals or is quite close to the mean, the standard score is zero or approximately to zero. This would cause a problem in calculating a ratio with this large variation, such as the ratio of L1 to L2 being negative or the ratio being invalid with zero as the denominator. 

In order to avoid the aforementioned extreme comparison cases, the principle is to make the ratio more meaningful and to enlarge the standard scores by moving a distance of a certain amount. The aim is to make the standardized data above zero with an augmented amount that is proportional to the original standard scores. That is, after the augmentation, the new minimum value should be right above zero and it should not be a too large value that is far over the original data’s digit place. Since it is known that the range between the minimum value and the maximum value of standard scores is fixed, the range is a good indicator to calculate the new minimum. 1/10 of the range is an appropriate value to represent the new minimum value that is larger than zero and proportionally at a similar scale comparable to the original minimum value. Then, the augmented amount is the difference between the new and old minimum value. With each original standard score plus this augmented amount, the data are more suitable for calculating the ratio. In the last step, ratios of L1 to L2 in language proficiency and that of L1 to L2 in preference were respectively calculated based on the standard scores with augmentation and then the final ratios were obtained by averaging them.

With the ratio as an index, the dominance of bilingualism (see Table 1 for participants’ descriptive information) is divided according to the principle that a ratio close to 1 indicates that the relative proficiency of individuals’ languages is balanced. Specifically, the final ratio of L1 to L2 was used as an indicator to rank all 93 bilingual individuals in a descending order. For the L1-dominant bilinguals, they were the top 31 bilinguals with a mean score (*M* = 1.28, *SD* = 0.13) of L1 to L2 ratio significantly (*t*(30) = 12.07, *p* < 0.001) larger than 1, while the bottom 31 participants were the L2-dominant bilinguals with a mean score (*M* = 0.87, *SD* = 0.13) of L1 to L2 ratio significantly (*t*(30) = −5.63, *p* < 0.001) below 1. Then, the remaining 31 participants were the balanced bilinguals with a mean score of ratio (*M* = 0.99, *SD* = 0.07) not significantly different (*t*(30) = −1.00, *p* = 0.321) from 1. The L1-dominant bilinguals, balanced bilinguals and L2-dominant bilinguals significantly differ from each other, *F* (2, 90) = 110.46, *p* < 0.001. 

### 2.2. Procedure and Materials

All participants received the test individually. The whole set of tests was composed of two conflict resolution tasks and one non-verbal intelligence test. Participants followed the same order of task administration with at first the flanker task and then the Simon task. They all completed the test of Raven’s Standard Progressive Matrices at the end. The stimuli in two cognitive control tasks were displayed via a Web technique in Google Chrome (a modern standard browser). The task design was based on the programming languages HTML5 and JavaScript. A server stored the response data into a MySQL database through the interface of the Ruby-on-Rails Application.

#### 2.2.1. Raven’s Progressive Matrices

Since IQ is the factor that has a high correlation with cognitive control ability [56], the measure of IQ was evaluated here as a control variable. A non-verbal intelligence evaluation was conducted through the standard version of Raven’s Progressive Matrices [57]. This Raven’s Matrices test is a measure of analytic reasoning and it consists of 60 matrices which are classed into 5 sets (A to E) containing 12 matrices for each ordered from a low to high difficulty level. A correct answer to each matric accounts for 1 point and the total score was 60. The mean IQ score of each language dominance group was at the same level with *F* (2, 90) = 3.065, *p* = 0.052. (see Table 1).

#### 2.2.2. Flanker Task

In this study, the Eriksen flanker [58] was implemented. The participants were presented with the stimuli constituting of 5 equally spaced arrows in a row on the screen and they were required to respond as quickly and accurately as possible to the direction where the central arrow points and to ignore the irrelevant flankers. In the congruent trials, the central arrows and the surrounding arrows point to the same direction (both leftward or rightward, e.g., → → → → →), while in the incongruent trials, the flankers are in an opposite direction to the central target arrow (e.g., → → ← → →). In the neutral trials, the surrounding arrows are not overlapped with a response (e.g., — — → — —). The trial presentation was that it started with a fixation of 500 ms followed by a blank interval of 250 ms and then a stimulus was displayed until the participant gave a response up to 2500 ms. A 250 ms blank interval was prior to the next trial. 12 practice trials were given to the participants and the test consisted of 126 trials with 42 congruent, 42 neutral and 42 incongruent trials. All trials were presented in a random order.

#### 2.2.3. Simon Task

In the Simon task [59], at one time one square coloured either in red or in blue was displayed on the screen. Participants were instructed to distinguish the colour of the stimulus and ignore the location of it. They pressed the key A (left side) on the keyboard when a red square was presented while key L (right side) was pressed when a blue square appeared. In the congruent trials the stimuli were presented at the same direction of the corresponding response, while in the incongruent trials the location of the stimuli was opposite to the target response. In the neutral trials, the stimuli were located in the centre. The event of presentation was as described in the flanker task. The number of experimental trials was 126 with an equal ratio for each trial condition.

## 3. Results

The analysis of the two cognition tasks was by reaction times (RT) and accuracy rates. The mean RTs of correct trials for all individual subjects was calculated and outlier RTs deviating from the mean by 2.5 standard deviation of the mean were excluded from the process of analyses. With this procedure, 2.25% flanker data was eliminated and 2.46% of the data in the Simon task was cut off. In the Simon task, the data of one participant in L1-dominant group was excluded from the analyses, because his rate of accuracy in incongruent trials was below 60%. As for the variable IQ, its mean for each bilingual group was at the margin of being different (*F* (2, 90) = 3.065, *p* = 0.052) and it was then taken into consideration as a covariate. Therefore, in order to measure the effect of language dominance types within bilinguals on cognitive control and to explore the differences of each pattern of conflict control tasks, 3 (Compatibility: congruency, neutral, incongruency) × 3 (Language Dominance Group: L1-dominant, balanced, L2-dominant) repeated measures ANCOVAs were used to examine the bilingual effect respectively on the Stimulus-Stimulus inhibition (flanker task) and the Stimulus-Response inhibition (Simon task). One-way ANCOVAs were conducted to examine if there were differences in conflict effect across the three groups of bilingual dominance. Furthermore, Pearson correlation analyses were applied on the same data to examine the trade-offs of reaction time and accuracy rates for each bilingual dominance group on the different conditions of trials and on the overall performance.

### 3.1. Flanker Task

For the two-way ANCOVA analysis of RTs (see Table 2 for descriptive statistics), the Mauchly’s test for sphericity assumption is significant (χ^2^(2) = 8.44, *p* < 0.05) and to correct the within-subjects tests, the Greenhouse-Geisser estimates of sphericity (ε = 0.92) were reported. Controlling for IQ, the effect of Compatibility was significant (*F*(1.83, 163.08) = 18.52, *p* < 0.001, η_p_^2^ = 0.172), indicating that reaction time to incongruent trials (*M* = 746 ms, *SD* = 89) was slower than to congruent trials (*M* = 682 ms, *SD* = 84) and to neutral trials (*M* = 670 ms, *SD* = 77). However, there was no main effect of Language Dominance Group (*F*(2, 89) = 1.77, *p* = 0.177, η_p_^2^ = 0.038), indicating that the overall RTs averaging over all levels in Compatibility were equivalent across the three language dominance groups. No two-way interaction between Compatibility and Language Dominance Group (*F*(3.67, 163.08) = 0.84, *p* = 0.496, η_p_^2^ = 0.018) was found. In the one-way ANCOVA analysis, response latencies to the flanker effect were similar across all three language dominance groups (*F*(2, 89) = 0.94, *p* = 0.396).

For the results of the accuracy rates (see Table 2), the Mauchly’s test indicated that the data were not spherical (χ^2^(2) = 76.87, *p* < 0.001) and the corrected results were used with reference to the Greenhouse-Geisser estimates of sphericity (ε = 0.63). After controlling for IQ in the analysis of accuracy rates in the flanker condition, it found no main effect of Compatibility (*F*(1.26, 112.48) = 0.02, *p* = 0.930, η_p_^2^ < 0.001), suggesting that there was no difference between incongruent trials (*M* = 96.42%, *SD* = 3.47) neutral trials (*M* = 99.41%, *SD* = 1.34) and congruent trials (*M* = 99.67%, *SD* = 0.90). There was no main effect of Language Dominance Group (*F*(2, 89) = 0.92, *p* = 0.401, η_p_^2^ = 0.020) and neither was the interaction between Compatibility and Language Dominance Group (*F*(2.53, 112.48) = 0.17, *p* = 0.887, η_p_^2^ = 0.004). In the one-way ANCOVA, the conflict effect incurred the similar pattern of accuracy rates across L1-dominant, balanced and L2-dominant bilinguals (*F*(2, 89) = 0.01, *p* = 0.988).

**Figure 1:**
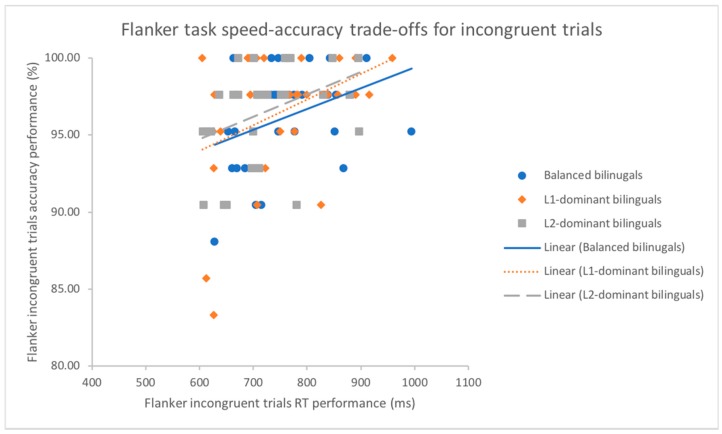
Scatter plot and regression fit lines demonstrating strategy of trading between mean response time (ms) and mean accuracy rates (%) for incongruent trials in the flanker task for L1-dominant, balanced and L2-dominant groups.

**Figure 2:**
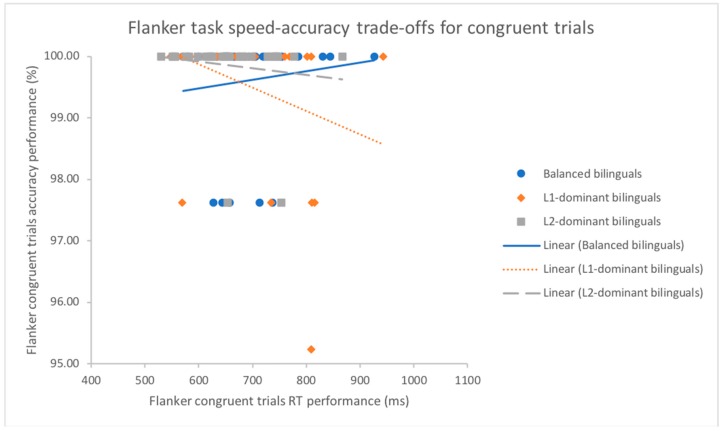
Scatter plot and regression fit lines demonstrating strategy of trading between mean response time (ms) and mean accuracy rates (%) for congruent trials in the flanker task for L1-dominant, balanced and L2-dominant groups.

Concerning the correlation between response time and accuracy rates, the results of Pearson’s correlational analysis showed that the speed-accuracy trade-offs in the aspect of overall performance across all conditions of Compatibility were significant for balanced bilinguals (*r*(31) = 0.36, *p* < 0.05) but not for L1-dominant bilinguals (*r*(31) = 0.24, *p* = 0.198) and L2-dominant bilinguals (*r*(31) = 0.35, *p*= 0.054) on the global performance. Furthermore, with an investigation of the speed and accuracy relationship with regard to the incongruent trials (see Figure 1), significant positive correlations were found for each subset of bilingual dominance groups with *r*(31) = 0.39, *p* < 0.05 for the L1-dominant, *r*(31) = 0.37, *p* < 0.05 for the balanced and *r*(31) = 0.39, *p* < 0.05 for the L2-dominant, suggesting that there was no within bilingual group differences in this flanker conflict. However, the same analysis in each bilingual dominance group respectively examined on the congruent trials (see Figure 2) and the neutral trials disclosed no correlations (all *p*s > 0.069). 

Pairwise comparisons between three dominance subsets of bilinguals were examined by using Fisher’s r to z transformation with the cocor calculator [60]. As for testing the correlation coefficient against each group in the overall performance, the results revealed that no significant difference was found between balanced and L1-dominant bilinguals (*z* = 0.494, *p* = 0.621), between balanced and L2-dominant bilinguals (*z* = 0.043, *p* = 0.966) and between L1-dominant and L2-dominant bilinguals (*z* = −0.452, *p* = 0.652). In the incongruent trials, the comparisons of *r* showed that there was no significant difference between balanced and L1-dominant bilinguals (*z* = −0.088, *p* = 0.930), between balanced and L2-dominant bilinguals (*z* = −0.088, *p* = 0.930) and between L1-dominant and L2-dominant bilinguals (*z* < 0.001, *p* = 1.000). In the congruent trials, there is a slight trend toward a significant difference between balanced and L1-dominant bilinguals (*z* = 1.692, *p* = 0.091). It was found that the differences in Pearson’s coefficient *r* was not significant between balanced and L2-dominant bilinguals (*z* = 0.979, *p* = 0.328) and between L1-dominant and L2-dominant bilinguals (*z* = −0.714, *p* = 0.476). In the neutral trials, there were no significant differences in the pairwise comparisons of *r* (all *p*s > 0.197).

### 3.2. Simon Task

In the RTs results (see Table 2) of the two-way ANCOVAs by controlling for IQ, a main effect of Compatibility, *F*(2, 176) = 8.90, *p* < 0.001, η_p_^2^ = 0.092, showed that responses to incongruent trials (*M* = 715 ms, *SD* = 86) were slower than to congruent trials (*M* = 678 ms, *SD* = 84) or to neutral trials (*M* = 693 ms, *SD* = 85), indicating that a Simon effect was found across all language dominance groups. However, there was no main effect of Language Dominance Group, *F*(2, 88) = 1.58, *p* = 0.212, η_p_^2^ = 0.035, indicating that there was no difference across the three language dominance groups on reaction times averaging over all conditions of Compatibility. No two-way interaction between Compatibility and Language Dominance Group was found, *F*(4, 176) = 0.54, *p* = 0.706, η_p_^2^ = 0.012. The one-way ANCOVA analysis yielded no significantly reduced Simon effect (*F*(2, 88) = 0.36, *p* = 0.701) across L1-dominant, balanced and L2-dominant bilinguals.

In terms of results in accuracy rates (see Table 2), the results revealed a significant difference (*F*(2, 176) = 3.87, *p* < 0.05, η_p_^2^ = 0.042) between each condition of Compatibility with confounding for IQ, suggesting that a higher accurate performance in congruent trials (*M* = 97.80%, *SD* = 2.98) was found compared to incongruent trials (*M* = 94.85%, *SD* = 3.93). The score in neutral trials (*M* = 97.67%, *SD* = 2.78) was similar to that in congruent trials and the lowest accuracy rate was found in incongruent trials. Similar to the results of RTs analysis, there was no main effect of Language Dominance Group (*F*(2, 88) = 0.81, *p* = 0.447, η_p_^2^ = 0.018) and neither was the interaction between Compatibility and Language Dominance Group (*F*(4, 176) = 0.17, *p* = 0.954, η_p_^2^ = 0.004). In the one-way ANCOVA analysis for conflict effect across each group the difference of accuracy performance between congruent and incongruent trials was similar across all language dominance groups, *F*(2, 88) = 0.19, *p* = 0.829.

**Figure 3:**
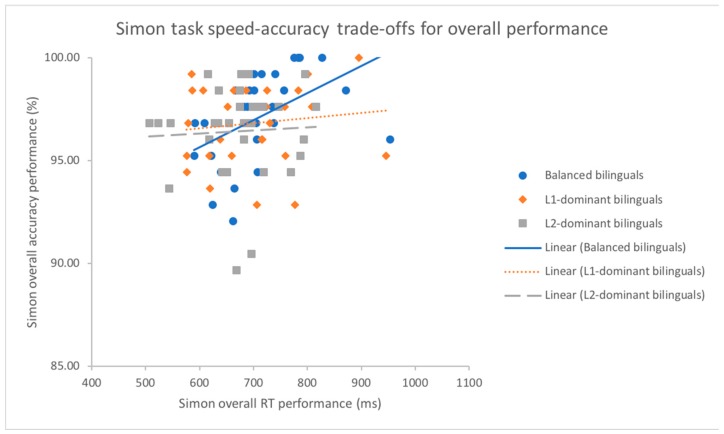
Scatter plot and regression fit lines demonstrating strategy of trading between mean response time (ms) and mean accuracy rates (%) for overall performance in the Simon task for L1-dominant, balanced and L2-dominant groups.

**Figure 4:**
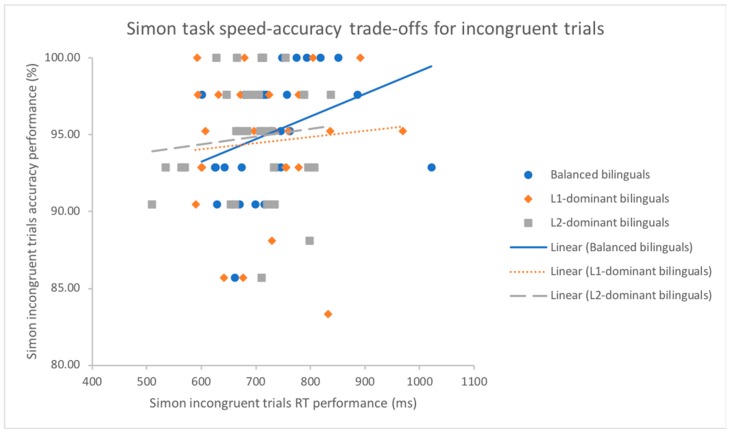
Scatter plot and regression fit lines demonstrating strategy of trading between mean response time (ms) and mean accuracy rates (%) for incongruent trials in the Simon task for L1-dominant, balanced and L2-dominant groups.

The analysis of Pearson’s correlation demonstrated that a trade-off between reaction speed and accuracy rates existed for the subgroup of balanced bilinguals (*r*(31) = 0.48, *p* < 0.01) on the global performance across all trials of Compatibility, whereas there was no significant correlation for the subgroups of L1-dominant (*r*(30) = 0.12, *p* = 0.519) and L2-dominant bilinguals (*r*(31) = 0.05, *p* = 0.782) (see Figure 3). A similar correlation pattern was found as well in congruent trials where slower reaction time was significantly correlated with higher accuracy rates for balanced bilinguals (*r*(31) = 0.43, *p* < 0.05) but not for the subsets of L1-dominant (*r*(30) = 0.11, *p* = 0.551) and L2-dominant bilinguals (*r*(31) = −0.06, *p* = 0.759). On the contrary, the analysis of Pearson’s correlation on each subgroup of bilingual dominance all indicated that there were no strong correlations between speed and accuracy for incongruent trials (see Figure 4) and neutral trials (all *p*s > 0.053).

To test the Pearson coefficient *r* against each dominance subset of bilinguals by using Fisher’s r to z transformation, the results for overall performance showed that no significant difference was found between balanced and L1-dominant bilinguals (*z* = 1.492, *p* = 0.136) and between L1-dominant and L2-dominant bilinguals (*z* = 0.262, *p* = 0.794). A nearly significant difference was found between balanced and L2-dominant bilinguals (*z* = 1.770, *p* = 0.077). The results of pairwise comparison of *r* in the congruent trials demonstrated that there was no significant difference between balanced and L1-dominant bilinguals (*z* = 1.296, *p* = 0.195) and between L1-dominant and L2-dominant bilinguals (*z* = 0.632, *p* = 0.527) and a nearly significant difference between balanced and L2-dominant bilinguals (*z* = 1.946, *p* = 0.052). The testing of Pearson’s *r* against each group in the incongruent trials showed that no significant difference was found between balanced and L1-dominant bilinguals (*z* = 1.020, *p* = 0.308), between L1-dominant and L2-dominant bilinguals (*z* = −0.037, *p* = 0.970) and between balanced and L2-dominant bilinguals (*z* = 0.992, *p* = 0.321). In the neutral trials, no significant differences of pairwise comparisons of Pearson’s *r* were found (all *p*s > 0.176).

## 4. Discussion

Our research examined the performance of L1-dominant, balanced and L2-dominant bilinguals on two types of cognitive control tasks. The sociolinguistic factor of languages acquired by bilinguals was controlled for, with a focus on the Uyghur-Chinese bilinguals whose native language is a regional language. The aim was to test the effect of bilingual dominance types on Stimulus-Stimulus and on Stimulus-Response cognition tasks, that is, the Simon task and the flanker task, especially with the investigation of the role of speed-accuracy trade-offs.

### 4.1. No Effect of Bilingual Dominance Types on Speed and Accuracy Performances in Cognitive Control Tasks

One of the findings in the present study was that bilingual dominance patterns had no effect on cognitive control in both the flanker and the Simon tasks with interpreting the results separately in terms of RT and accuracy rates. More specifically speaking, there was no evidence showing that degrees of bilingual dominance moderated the specific performance in the incompatibility trials, that is, the control of interference suppression or the overall performance by examining all trial conditions as a whole, that is, the control of monitoring (similar measures for cognitive performance, see for example in Costa et al. [9]). Since there were no response time differences across the three dominance subsets of bilinguals, the findings supported neither our hypothesis nor the controlled-dose hypothesis. Although this finding shows no support for our first hypothesis that balanced bilinguals outperform dominant bilinguals in each conflict resolution task, it is partly in line with the previous evidence that the bilingual dominance patterns had no effect on interference suppression in the flanker task [35,38]. However, it should be noted that the results for the flanker type inhibition task were contradictory with each other in the previous findings of Gathercole et al. [37] research supporting a better performance in interference suppression from balanced bilinguals relative to the dominant ones. On the other hand, our results for the Simon task are inconsistent with preceding studies [15] and the present study fails to confirm that performances in conflict inhibition were differentiated by degree of bilingual dominance. In general, the pattern of our results revealed no effects of bilingualism on the non-linguistic cognitive control tasks and this is in line with the meta-analytic review by Lehtonen et al. [13]. Furthermore, in a new study by Hilchey et al. [61], they disconfirmed their previous claims in the earlier review study [45] and found no evidence for a transfer from bilingualism to the nonverbal inhibitory control and monitoring. To be noted, Paap [62] pointed out that the overall RT that the average across conflict and non-conflict trials is an impure measure of monitoring and a block of baseline composed of congruent trials should be combined with the mixing block to purely measure monitoring.

One potential account for the absence of balanced bilinguals’ better performance in inhibitory control is the high automaticity of intra-lingual and inter-lingual knowledge in two languages [15,35]. Following the framework of language processing models [63], the interference control of within and across language activation are present in bilingual language recognition and production processes and it is proposed that the cross-linguistic knowledge is less strong than the within language knowledge [15]. However, when balanced bilinguals develop a more automatized and strengthened link across languages, little inhibitory control is required resulting in a ceiling level in executive control and no transfer effect. Furthermore, it is reasoned that the sociolinguistic factor of language switching frequency plays a role in the cognitive advantage in that code-switching increases the link to inhibitory control [64]. For the dominant bilinguals being tested in the present study, 61% out of them reported in the LEAP-Q questionnaires that they were immersed in a monolingual community either dominant in Uyghur or in Chinese during childhood and teenage years. It indicates that the majority of L1 or L2 dominant bilinguals lack the capacity of exercising the inhibitory control in language processing. This could explain why there was no effect of bilingual dominance on both cognitive control tasks detected in the present research.

### 4.2. Differences of Speed-Accuracy Trade-Offs in Bilingual Dominance Type

Another further exploration about the effect of within-bilinguals’ dominance on each cognitive control task in the present study lies in the investigation of each subset of bilinguals’ resolution strategy towards speed and accuracy. The result is consistent with our prediction that balanced bilinguals as opposed to dominant bilinguals show a clear task strategy with a significant speed-accuracy trade-off in both flanker and Simon tasks. More critically, the finding of this strategy of either favouring response speed over accurate choice or emphasizing the accuracy at the cost of speed is only present in the global performance across all compatibility conditions where the overall reaction to congruent and neutral trials in addition to incongruent trials were mixed.

Our findings about the speed-accuracy trade-off strategy exploited by balanced and L1- or L2-dominant bilinguals across flanker and Simon tasks build on the Struys et al.’s [51] study which revealed that only bilingual children demonstrated a significant trade-off strategy across both tasks compared to monolinguals. In the present study, the strength of correlation between speed and accuracy is only significant within the group of balanced bilinguals but the comparison of the coefficient r in balanced bilinguals with that in L1- or L2- dominant bilinguals showed only marginally significant pairwise differences. The reason for more subtle between-group differences in the current study compared to Struys et al. [51] may be that the current study investigated strategic differences within groups of bilinguals, while the one by Struys et al. [51] compared bilinguals to monolinguals. However, the present findings suggest that potential variations between bilinguals and monolinguals are driven by bilingual within-group differences related to language dominance types. 

A possible reason for these differences within bilinguals is that balanced bilinguals vary from L1-or L2-dominant bilinguals in that they experience more language conflict because of the equal strength of representations in both languages. Because of these highly competing responses, both bilinguals when using L1 and L2, may be expected to experience a higher degree of language conflict in the process of language selection compared to dominant bilinguals, who mainly experience language conflict during non-dominant language processing. Therefore, to solve this demanding linguistic conflict, balanced bilinguals need to develop a strategy to efficiently switch between the equal activation of each language tag at the lexical level [65,66]. We propose that the daily management of language conflict in balanced bilinguals may transfer into a subtle difference with other bilinguals in domain-general cognitive control, analogous to a lesser extent with the similar differences previously detected between bilingual and monolingual children [51]. 

Furthermore, the strategy of trading off between speed and accuracy was a clear pattern shown in the subset of balanced bilinguals in overall performance across both tasks. In the present study, the flanker task and the Simon task both constitute a randomized and mixing context where the proportion of congruent and incongruent trials is equally distributed. According to Costa et al.’s [3] study, this type of mixing context induces a maximum recruitment of the conflict monitoring system which refers to the cognitive ability to assess the probability of an upcoming conflict and to adjust the cognitive control system accordingly [67]. Specifically, before an actual conflict resolution comes into use in the flanker or Simon tasks, a constant monitoring is needed for attending to the presence or absence of conflict that occur with an equal probability. The high demands on the monitoring system related to overall performance on the flanker and Simon tasks may bear some similarity to the high-monitoring code-switching context in balanced bilinguals. Especially for balanced bilinguals, it is demanding for them to constantly attend to a potential upcoming switch to the other language that shares an equal strength of representation as the one in use. When balanced bilinguals are confronted with the cognitive control tasks featured with mixing a similar probability of presenting conflict and non-conflict trials, balanced bilinguals may transfer the strategy trained in language selection of two languages with similar strengths of representation to strategically cope with the non-linguistic cognition domain with a high-monitoring context.

### 4.3. Strategy Differences in Stimulus-Stimulus and Stimulus-Response Inhibition Tasks

The second research interest was about whether the cognitive performance of bilinguals in two different types of inhibitory control tasks differentiate in terms of the application of a strategy in speed-accuracy trade-offs. Generally speaking, we expected to observe a particular pattern between speed and accuracy in the Stimulus-Stimulus task for all dominance types of bilinguals but not in the Stimulus-Response task. The findings partially confirm our hypothesis with the evidence that both balanced and dominant bilinguals demonstrate a strategy in the flanker task, whereas the findings are inconsistent with our prediction, in that balanced bilinguals show a speed and accuracy trade-off strategy in the Simon task. More specifically speaking, individual bilinguals exploit a clear pattern of strategy to optimize the conflict resolution in the executive functioning and when encountered with the language conflict, they may strategically optimize interference resolution to language competition.

It is worth noting that at the level of incongruent trials, there is a clear contrast between the flanker and the Simon task. That is, the whole bilingual test population approach the Stimulus-Response inhibition, that is, the Simon task, without trading between speed and accuracy. One reason is that these strategy differences are caused by the underlying mechanism in bilingual language processing. For the bilingual language recognition process, in the framework of IC computational bilingual model (for a review of empirical evidence, see [5,68]), the Stimulus-Stimulus inhibition (flanker conflict) mechanism is analogous to the bilingual processing context of language recognition where perceptually similar lexical candidates of target language and distractor language are co-activated. More specifically, the cross-language interference arises with the presence of overlap between the activated lemmas of the relevant language and the interference from the co-activated lexical representations of the non-target language in word recognition. In the process of language comprehension, it shares a highly similar mechanism underlying the Stimulus-Stimulus inhibition.

In the same vein, the language production process of bilinguals involves more than the competition of language candidates at the lexical and phonological level, since speech planning and behavioural responses are additionally required [69]. The empirical results (e.g., [70] ) reveal that the Stimulus-Stimulus competition (flanker conflict) is present in the bilingual speech production in that similar phonologies of distractor language and the target word sounds are both triggered to compete for output. Besides, different naming responses from both languages simultaneously arise to generate a cross-language competition for response selection in the process of production and this Stimulus-Response inhibition (Simon conflict) is found to be much more involved in the language switching context [48].

It is indicative that the presence of a Stimulus-Stimulus inhibitory mechanism is found in both the language recognition and production process of bilinguals, whereas Stimulus-Response competition is exclusively present in the speech production context with the competition of co-activated response options from two languages. Therefore, we propose that bilinguals are more sensitive to Stimulus-Stimulus cognitive control tasks and that their bilingual experience enables them to form either a speeding up strategy or a choice focused on accuracy in the most frequently involved Stimulus-Stimulus inhibition. In addition to previous studies suggesting a more efficient bilingual performance in the Stroop task than the bilingual Simon task (e.g., [50] ), we extend the finding further to the investigation of a strategy pattern underlying each type of task by a within-bilingual comparison across different dominance types. Interestingly, it was found that the length of reaction times in the flanker effect is larger than in the Simon task and thus to some extent we as well add evidence to the result that Stimulus-Stimulus inhibition and Stimulus-Response inhibition cause different behavioural effects [55]. Most importantly, the findings in the present study indicate that across bilinguals with different relative proficiency of two languages, they all more proficiently develop a strategy of trading between speed and accuracy in the Stimulus-Stimulus (flanker type) inhibition which to a large extent accounts for the underlying mechanism of bilinguals’ language interference control as well rather than in the Stimulus-Response (Simon type) situation.

As for the finding that balanced bilinguals implement a strategy in speed and accuracy in congruent conditions in the Simon task, this evidence is not consistent with our prediction of no transfer of bilinguals’ speed and accuracy trade-off strategy to Stimulus-Response inhibition. Since it is in the Simon non-conflict trials that the balanced bilinguals’ strategy was detected, it means the irrelevant interference at the response level is absent. The condition with non-conflict trials in the Simon task is similar to the unilingual language production context where there is also an absence of Stimulus-Response competition [71]. Thus, we assume that this subset of balanced bilingual individuals compared to dominant bilinguals are more frequently exposed to the unilingual processing context and this bilingual experience enhances the strategy in a non-conflict Stimulus-Response context.

## 5. Conclusions

To sum up, one of the major findings in the present study is that the within-bilingual factor, that is, language dominance type, has no explicit effect on the performance of cognitive control tasks and the advantage of balanced bilinguals is not present in the separate analysis of speed and accuracy. However, by examining the strategy tendency, it uncovers that the transferred cognitive control differences between each subset group of bilinguals primarily lies in the goal maintenance and monitoring process. Another principle finding is that the comparison of the flanker and the Simon tasks by investigating the speed-accuracy trade-offs indicates that regardless of the degrees of bilingual proficiency, the underlying mechanism of bilingual language inhibitory control is to a large extent dependent on the type of Stimulus-Stimulus conflict resolution that is present in both language recognition and production processes. This finding implies that being exposed to different sociolinguistic contexts where different types of inhibition are induced, such as Stimulus-Stimulus or Stimulus-Response conflicts may lead to various patterns in strategic task tendencies in bilingual cognitive processing.

## Figures and Tables

**Table 1:** Demographic information of bilingual language dominance groups.

Bilingual Groups			
	Uyghur (L1) dominant	Chinese (L2) dominant	Uyghur-Chinese Balanced
Age	19.61 (1.38)	19.23 (0.92)	19.94 (1.63)
Number	31	31	31
Male/Female	13/18	12/19	9/22
Raven IQ score	44.45 (4.88)	47.03 (4.74)	47.32 (5.43)
Proficiency ratio of L1/L2 ^1^	1.27 (0.30)	0.91 (0.12)	1.06 (0.22)
Preference ratio of L1/L2	1.52 (0.37)	0.81 (0.25)	0.97 (0.21)
Ratio of balance ^2^	1.28 (0.13)	0.87 (0.13)	0.99 (0.07)

Standard deviations are in parentheses. ^1^ Proficiency ratios of L1/L2 (and Preference ratio of L1/L2) are calculated based on the formulated standardized data. ^2^ Ratio of balance, an average score of the previous two ratios, is the indicator to divide dominance groups. That the ratio is close to 1 indicates the relative proficiency of individuals’ languages is balanced.

**Table 2:** Mean response times in milliseconds and accuracy in percentages with standard deviation in parentheses for flanker and Simon tasks by bilingual dominance groups and trial conditions.

			Congruent	Neutral	Incongruent
RT	flanker task	L1	686 (95)	668 (83)	750 (98)
		L2	663 (77)	652 (68)	724 (82)
		Balanced	697 (78)	688 (77)	766 (86)
		L1	683 (90)	702 (96)	719 (95)
	Simon task	L2	657 (77)	672 (84)	694 (77)
		Balanced	694 (83)	705 (74)	731 (85)
Accuracy	flanker task	L1	99.54 (1.14)	99.46 (1.33)	96.47 (4.21)
		L2	99.85 (0.59)	99.77 (0.72)	96.55 (3.06)
		Balanced	99.62 (0.89)	99.00 (1.71)	96.24 (3.12)
	Simon task	L1	98.10 (2.89)	97.86 (2.89)	94.53 (4.43)
		L2	97.24 (2.95)	97.16 (3.22)	94.86 (3.89)
		Balanced	98.08 (3.10)	98.00 (2.14)	95.16 (3.56)
